# Compliance of community teams with specialist service recommendations for obsessive–compulsive and body dysmorphic disorders

**DOI:** 10.1192/pb.bp.115.052530

**Published:** 2016-10

**Authors:** Paul M. Harris, Lynne M. Drummond

**Affiliations:** 1 South West London and St George's Mental Health NHS Trust

## Abstract

**Aims and method** To examine how often referring community mental health teams (CMHTs) utilised treatment recommendations made by the national highly specialised service for patients with severe obsessive–compulsive disorder (OCD) and body dysmorphic disorder (BDD). We analysed all patient notes for admissions to the unit (August 2012–August 2014) and recorded how many treatment recommendations were implemented by CMHTs prior to admission and at 6 months post-discharge.

**Results** Overall, 66% of our recommendations were met by CMHTs prior to admission and 74% after discharge. Most recommendations concerned medication and the continued need for care coordination by the CMHT.

**Clinical implications** A significant proportion of patients in our audit did not receive optimum treatment in the community as recommended by our service. As highly specialised services are a limited resource and these patients have not responded to previous treatment, this has implications for the use of such resources.

The current guidelines from the National Institute for Health and Care Excellence (NICE) for treatment of obsessive–compulsive disorder (OCD) and body dysmorphic disorder (BDD) in England and Wales outline a stepped six-stage care model approach, with each step related to the severity of the individual's illness.^1^ The sixth stage of this care model, for the most unwell patients, advocates the utilisation of specialist intensive treatment or in-patient services, and states that these services should be provided by multidisciplinary teams with specific expertise in this area. Patients who require in-patient treatment may be at risk of death or severe personal neglect.

In recent years community mental health teams (CMHTs) have faced increasing and widening pressures. The provision of community psychiatric services has been subject to reorganisation and change; for example, specialist assessment and recovery teams have replaced previous models of working. This restructuring has had an impact on continuity of care. Indeed, it has been argued that the adoption of new models of psychiatric care in the community, and the growing number of specialist teams, has led to the fragmentation of services and a poorer experience for both patients and professionals.^2^ In some instances these new systems have been associated with reduced continuity of care, with patients encountering a greater number of health professionals who are unable to develop meaningful and prolonged relationships with them owing to brief and changing contact.^3^

The recent cut in in-patient beds has also placed increasing pressure on community teams. Indeed, there were concerns within our service that local teams are finding it increasingly difficult to implement our advice for their severely unwell patients and those whose illness is treatment refractory. Members of our team felt this was occurring both prior to the patients' planned admission and after discharge. In light of these concerns, and being mindful of recent changes in service provision, we chose to examine the number of treatment recommendations made by our team that had been implemented by CMHTs prior to admission and then again at 6 months post-discharge from the ward.

## Method

### Setting

There are currently only a few centres in the UK that provide specialist intensive care of severe and treatment-refractory OCD and BDD and all of these are based in the south-east of England.^4^ There is only one 24-h-staffed in-patient service, based at South West London and St George's Mental Health NHS Trust. This in-patient service, funded by NHS England Highly Specialised Services Commissioners (NHS England HSS), is a 14-bed unit (it has been described by Boschen *et al*^5^). Highly specialised services are funded directly by NHS England and the expected demand is fewer than 500 patients per annum. Examples of other services include liver transplantation, enzyme replacement therapy, secure forensic mental health services for young people as well as services for profound, refractory OCD and BDD. The specialist unit in London comprises psychiatrists (from training grade to consultant level), specialist cognitive–behavioural therapy (CBT) therapists, nursing staff, healthcare assistants, occupational therapists and allied therapy staff. It is the only 24-h-staffed unit for OCD/BDD in the NHS, and thus concentrates on treating those who not only have failed all previous treatments, but who could not be safely treated in less intensive services. The unit accepts referrals from local CMHTs throughout England when the referral criteria have been met (see the Discussion section). Patients who meet these criteria are invited to have an initial assessment by a member of the specialist team to evaluate whether they are suitable for treatment under the auspices of NHS England HSS OCD/BDD Services. If this is ascertained then a decision is made as to the most suitable service to be offered. These range from out-patient and intensive home-based treatments through to the 24-h-staffed unit. Consequently, only those patients with the most profound disorders who are unable to manage in any less supported environments are admitted. Patients from Scotland, Wales or Northern Ireland are still referred via their CMHTs but in these cases funding needs to be secured via their local arrangements. Common reasons for recommending 24-h-staffed in-patient treatment include:

- the patient is a danger to themselves – this is most commonly owing to extreme self-neglect resulting in, for example, extreme dehydration
- the patient is unable to carry out activities of daily living without interventions from others – for example, almost a half of patients admitted to the service are incontinent of urine or faeces on admission
- there is a an additional diagnosis as well as OCD or BDD requiring 24-h observation – for example, a recent history of unstable eating disorder or recent history of substance misuse
- they have failed to respond to treatment in other less intensive, highly specialised services.

Patients that meet our referral criteria are offered an initial assessment. The findings of this assessment are discussed at a meeting of the multidisciplinary team (MDT), and a care plan, with specific treatment recommendations, is developed. The plan is sent to the patient and their general practitioner (GP), as well as to the referring team. If the patient is suitable for an in-patient admission it is requested that the treatment recommendations are put in place by their local team before they are admitted to the unit.

An in-patient admission typically lasts for 4 to 6 months, and on discharge another care plan, with individual recommendations, is sent to the patient, their GP and referring team. The patient is then followed up by the national service at 1 week, 1 month, 3 months, 6 months and 1 year. The responsibility for continuity of care, and ongoing rehabilitation, is dependent on the active involvement of the CMHT.

### Analysis

We scrutinised the case notes of all patients who were admitted to our in-patient ward between August 2012 and August 2014.

First, using the initial assessment report sent to the referring CMHT, we recorded how many separate care plan recommendations our team had made. These were recorded by type: medication related, care coordination, physical health intervention, and other. We then examined the notes from when the patient was first admitted to our ward (admission clerking, GP records, and other correspondence) to establish how many of our recommendations had been met. Second, using the discharge care plan sent to the local CMHT, we again recorded how many recommendations our team had made by type. We then analysed the notes from our follow-up appointments to see how many of these recommendations had been met. Only patients who had a minimum of 6 months' follow-up were included in this analysis.

## Results

A total of 52 patients were included in the study, of whom 32 (62%) were male and 20 (38%) were female. The mean age was 42.4 years (s.d. = 12.8). The mean Yale-Brown Obsessive Compulsive Scale (YBOCS) score pre-admission was 36.19 (s.d. = 3.08) and at the end of admission 24.73 (s.d. = 8.92). The mean age at onset of OCD symptoms was 22.04 years (s.d. = 9.93).

After the initial out-patient assessment of the sample of 52 patients, a total of 99 care plan recommendations were made, 65 (66%) of which had been met prior to admission to our ward. Most of the recommendations focused on the need for continued review by the CMHT with allocation of a care coordinator. This recommendation was made 50 times and was met 39 times (78%). Our team also made 41 medication-related recommendations (substitutions, adjuncts, increases or decreases in dose in line with NICE guidelines), but only 25 (61%) of these changes were made prior to admission. For most patients (*n* = 40) medication-related and care coordination-/increased CMHT involvement-related recommendations were made together. All recommendations by type can be seen in Table 1.

**Table 1:** Treatment recommendations made to CMHT by the specialised unit

	Prior to admission^a^		After discharge min. 6 months follow-up^b^	
Type of recommendation	*N*	Achieved *n* (%)	*N*	Achieved *n* (%)
Medication-related	41	25 (61)	36	28 (78)

Care coordination/increased CMHT involvement	50	39 (78)	40	31 (78)

Physical health intervention	5	0(0)	4	1 (25)

Other specialist referral (e.g. drug and alcohol services)	3	1 (33)	8	5(63)

Total	99	65 (66)	88	65 (74)

CMHT, community mental health team.

a. *n* = 52 admissions.

b. *n* = 39 patients.

c. *n* = 10 patients.

After discharge 39 of the 52 admissions (71%) had at least 6 months of recorded follow-up from our team. For these patients, we made a total of 88 treatment recommendations at discharge and there was evidence that 65 (74%) of these were met by the local CMHT at 6 months. The types of recommendations can be seen in Table 1. Ten patients had follow-up of 3 months only, and our team made a total of 27 recommendations for them; only 10 of these were met (37%) (Table 1). Of note, our team requested that all of these patients have a care coordinator.

Patients were grouped by response to treatment. Those demonstrating a greater than 35% improvement in YBOCS score were categorised as responders, those showing improvement of between 25 and 35% were grouped as partial responders, and those demonstrating a less than 25% improvement were categorised as non-responders (Table 2).

**Table 2:** Responders, partial responders and non-responders to treatment

	*n* (%)
Responder	25 (48)

Partial responder	6 (12)

Non-responder	21 (40)

Analysis of these groups showed no relationship between patients who did well in treatment (Table 2) at the national OCD/BDD unit and whether or not the CMHT was more or less compliant with our treatment recommendations (Pearson correlation coefficient 0.147, *P* = 0.299). Pearson correlation coefficient was used to analyse these data as the variables were normally distributed.

## Discussion

It has been estimated that approximately 1% of the European population has clinically relevant OCD.^6^ Modern treatment of this disorder can be very effective, with improvement in symptoms following graded exposure therapy seen in an estimated 75% of patients^7^ and 60% responding to SSRI medication.^8^ However, in a proportion of patients the OCD remains treatment refractory and such patients may need more specialist and intensive input.

In 2007, it was decided that, owing to the lack of highly specialised services for OCD/BDD nationally, these should be funded centrally. After various reorganisations in healthcare provision and commissioning, this is currently funded by NHS England HSS. When this commissioning group was established it was agreed to operationalise the criteria for defining a patient as falling into the stage six (most severe disorder) category as:

- YBOCS score >30/40 (or equivalent on the YBOCS-BDD scale)
- failure to respond to two previous trials of serotonin reuptake inhibiting (SRI) drugs in maximum *British National Formulary* (BNF)-approved dosages for a minimum of 3 months each
- failure to respond to further recognised psychopharmacological interventions for refractory OCD (e.g. addition of a dopamine-blocking agent to the SRI)
- failure to respond to two courses of CBT involving graded exposure and self-imposed response prevention (one of these courses should normally have taken place in the home environment or wherever the symptoms are maximal).

In recent years there have been increasing pressures on both local CMHTs and in-patient services, including a reduced number of in-patient beds and service reorganisations. With these considerations in mind, our evaluation provides some interesting findings.

After initial assessment, just over two-thirds of all recommendations (66%) made by our team were met. However, recommendations related to care coordination were met on just over three-quarters of occasions (78%), whereas medication-related recommendations were met less frequently (61%). Interestingly, after discharge adherence to medication-related recommendations was higher (78%), possibly as patients experienced the benefits of prolonged consistent medication at suitable dosages.

Patients who are eventually referred to highly specialised services on average have waited 20 years from initial diagnosis to accessing these services.^4,5,9–11^ Although they generally improve with our intervention, they are likely to have ongoing difficulties in some areas. This appears to be best managed through ongoing help from local mental health services as such patients are likely to need continuing support in various aspects of their lives in addition to ongoing medication and support for their OCD or BDD. Key to enabling earlier access, and continued adherence, to treatment is care coordination, which facilitates continuity of care and the development of trust and understanding between patients and their care team. The findings from our analysis, although encouraging, show that almost a quarter of all patients were not receiving care coordination. It is possible that this finding could be linked to changes in local services, and the increased pressures they face in the current financial climate.

In view of the delay for patients in accessing specialised services, it is important to note that treatment of even the most severely affected patients with OCD is remarkably successful, with improvements in YBOCS scores of up to 33% from baseline in this sample.^4,5,9–11^ After the initial assessment, local teams appear reluctant to implement medication-related recommendations. This could perhaps reflect confidence issues around prescribing high-dose SSRIs or augmenting SSRIs with low-dose antipsychotics. However, after discharge the continuation of prescribed medications was more likely to be met by community teams despite a significant proportion of patients being prescribed SSRIs at above the BNF-recommended maximum dose.

It is difficult to know how highly specialised services can help with improving confidence. CMHTs are advised that the service is happy to discuss any problems they may have with patients at any time and that the team's consultant psychiatrist is available for advice concerning medication. We also provide leaflets outlining psychopharmacological treatments for these conditions.

It is also interesting to note that the compliance by CMHTs with our treatment recommendations after discharge was not affected by patient response to in-patient treatment (Table 2). This emphasises the importance of continued CMHT care as all of the patients in our audit had comparable compliance rates regardless of the severity of their OCD symptoms at discharge. CMHT compliance rates post-discharge cannot be predicted by patient treatment response on the unit. Despite our expectation that it may be high-dose SSRI prescribing which would be a cause of concern for the CMHTs, in fact the majority (78%) complied with these recommendations.

## Conclusions

Although the results of the audit were better than might be expected, there is still much room for improvement, particularly as our service represents such a scarce resource. The question whether these rates have been negatively affected by recent changes to care is one that cannot be answered directly by this evaluation. However, the results do show that a large proportion of profoundly unwell patients are still not receiving what could be considered optimal treatment when in the community. This may be because of many factors, perhaps not least the fact that those with the greatest need are often most difficult to engage with. However, in an attempt to improve outcomes and collaborative working between our service and CMHTs, we encourage local care coordinators to play an active part in the treatment process, both at initial assessment and before discharge. For example, CMHT members are invited to attend discharge meetings at our unit.

We welcome debate and discussion as to how our service can help and support the CMHTs to ensure the ongoing support necessary for these patients.
